# Modelling and numerical computation for flow of micropolar fluid towards an exponential curved surface: a Keller box method

**DOI:** 10.1038/s41598-021-95859-x

**Published:** 2021-08-11

**Authors:** Qiu-Hong Shi, Tayyaba Shabbir, M. Mushtaq, M. Ijaz Khan, Zahir Shah, Poom Kumam

**Affiliations:** 1 Department of Mathematics, Huzhou University, Huzhou, 313000 People’s Republic of China; 2 Department of Mathematics, COMSATS University Islamabad, Park Road, Chak Shahzad, Islamabad, 44000 Pakistan; 3 Department of Mathematics and Statistics, Riphah International University I-14, Islamabad, 44000 Pakistan; 4 Department of Mathematical Sciences, University of Lakki Marwat, Lakki Marwat, 28420 Khyber Pakhtunkhwa Pakistan; 5 Center of Excellence in Theoretical and Computational Science (TaCS‑CoE), Faculty of Science, King Mongkut’s University of Technology Thonburi (KMUTT), 126 Pracha Uthit Rd., Bang Mod, Thung Khru, Bangkok, 10140 Thailand; 6 Fixed Point Research Laboratory, Fixed Point Theory and Applications Research Group, Center of Excellence in Theoretical and Computational Science (TaCS‑CoE), Faculty of Science, King Mongkut’s University of Technology Thonburi (KMUTT), 126 Pracha Uthit Rd., Bang Mod Thung Khru, Bangkok, 10140 Thailand; 7 Department of Medical Research, China Medical University Hospital, China Medical University, Taichung, 40402 Taiwan

**Keywords:** Engineering, Mathematics and computing

## Abstract

The numerical analysis of MHD boundary layer non-Newtonian micropolar fluid due to an exponentially curved stretching sheet is developed in this study. In the energy equation effects of viscous dissipation are included. For the mathematical description of the governing equations curvilinear coordinates are used. By utilizing exponential similarity variables, the modelled partial differential equations (PDEs) are reduced into ordinary ones. The resultant non-linear ODEs are numerically solved with two methods shooting and Keller box method. The study reveals that the governing parameters, namely, radius of curvature, material parameter, magnetic parameter, Prandtl number and Eckert number have major effects on the fluid velocity, micro-rotation velocity, surface friction, couple stress and heat transfer rate. The results indicate that the magnetic field diminishes the fluid velocity inside the hydrodynamics boundary layer whereas it enhances the temperature inside the thermal boundary layer. Microrotation profile decreases near the surface, as the magnetic parameter and radius of curvature increases but far away behavior is opposite. The material parameter enhances the velocity and microrotation profile whereas, opposite behaviors is noticed for the temperature distribution. Obtained outcomes are also compared with the existing literature and the comparison shows a good agreement with existing studies.

## Introduction

The stretching sheets due to heat transfer and boundary layer flow fascinated the researchers and the engineers due to its vast number of applications in industry such as liquid composite molding, wire drawing, metal spinning, extrusion of polymer sheets, gas blowing, manufacturing of plastic films, hot rolling and many more.

Sakiadis^1^ reported the effects of constant velocity over a solid wall. Tsou et al.^2^ examined the characteristics of heat transfer onto a stretching sheet. Analytical solution regarding the viscous fluid flow prompted by a linearly stretching surface was scrutinized by Crane^3^. Gupta and Gupta^4^ analyzed the effects of linear velocity over a stretchable sheet by taking into account the suction/blowing. Grubka and Bobba^5^ considered the heat transfer characteristic by taking into consideration the linear velocity with variable temperature distribution. By considering exponential velocity and temperature distribution Magyari and Keller^6^ firstly examined the flow behavior and heat transfer characteristics over a stretchable surface. Elbashbeshy^7^ carry forward the work of Magyari and Keller by considering the influence of suction and blowing on the surface. The characteristics of viscoelastic fluid regarding an exponential stretchable sheet was studied by Khan^8^. Ishak^9^ and Bidin and Nazar^10^ analyzed the boundary layer viscous fluid along a stretched surface with an exponential velocity under the influence of thermal radiations. Mass transfer towards an exponentially stretchable porous sheet was presented by Mukhopadhyay et al.^11^. Raju et al.^12^ worked on the flow features of Casson fluid over an exponential stretching with permeability. They also accorded the effects of chemical reaction, viscous dissipation, heat source and magnetic field. For other related works on flow due to stretching surface, the following references^13–18^ can be referred.

The classical hydrodynamics of Naiver Stoke model are not capable to describe the flow behavior of microstructure fluids viz; liquid crystals, polymeric suspension and animal blood. Physically micropolar fluids correspond to the fluids having randomly oriented (spherical), rigid micro-particles of different shape in a viscous medium, where these particles deformation is not examined. The micropolar fluids theory has been presented by Erigen^19,20^. Peddieson and McNitt^21^ numerically examined the boundary layer flow by considering the micropolar fluid model. Rosali et al.^22^ found the solution of boundary driven micropolar fluid model over a shrinking/stretching surface. Mandal and Mukhopadhyay^23^ reported the micropolar fluid generated by a stretchable exponentially sheet in the presence of moving free stream.

The stretching of curved surface has gained much attraction because of its mathematical interest for solving nonlinear equations in curvilinear coordinates. Sajid et al.^24^ pioneered the effects of linear velocity over a curved surface. Heat transfer mechanism over a curved stretched sheet along with a linear velocity was scrutinized by Abbas et al.^25^. The concept of suction and injection over a curved unsteady shrinking/stretching surface was incorporated by Rosa and Pop^26^. Sajid et al.^27^ documented the non-Newtonian micropolar fluid flow generated by the curved surface. Naveed et al.^28^ further extended the problem by adding the effects of thermal radiation. Hayat et al.^29^ pointed out the effects of MHD and homogenous-heterogenous reaction respectively in the flow of micropolar fluid along a curved stretched wall. Saleha et al.^30^ examined the time-dependent micropolar fluid towards a linearly stretching porous wall. All these investigations were made for the linear velocity over a curved surface. The effects of non-linear (power-law) velocity over the curved stretched surface were given by Sanni et al.^31^ By considering the effects of power law velocity Hayat et al.^32^ analyzed the numerical computation of nanofluid over a curved stretching sheet. Okechi et al.^33^ initiated the flow over a curved surface by taking into consideration the exponential similarity variables and velocity. Hayat et al.^34^ performed the characteristics of Darcy-Forchheimer flow of nanofluid towards a curved stretchable geometry with exponential velocity and temperature. Kamar et al.^35^ studied the problem of Casson fluid in the geometry of exponentially stretched curved surface under the influence of thermal radiation. Reddy et al.^36^ analyzed the dual solution for a non-Newtonian nanofluid flow through a curved surface by taking into consideration of Soret and Dufour effects.

In this novel research work, steady, incompressible flow of non-Newtonian fluid (micropolar fluid) is addressed over a stretched curved surface. Viscous effects is accounted. The governing flow expression are first altered into ordinary system and then computational results are computed. The main concern here to compute the numerical results through highly valuable numerical technique Keller box method and Runge–Kutta based Shooting Method. Numerical solution of the velocity, micro-rotation velocity, temperature profile, couple stress, skin friction coefficient, and Nusselt number are calculated numerically and presented graphically.

## Mathematical formulation

**Figure 1:**
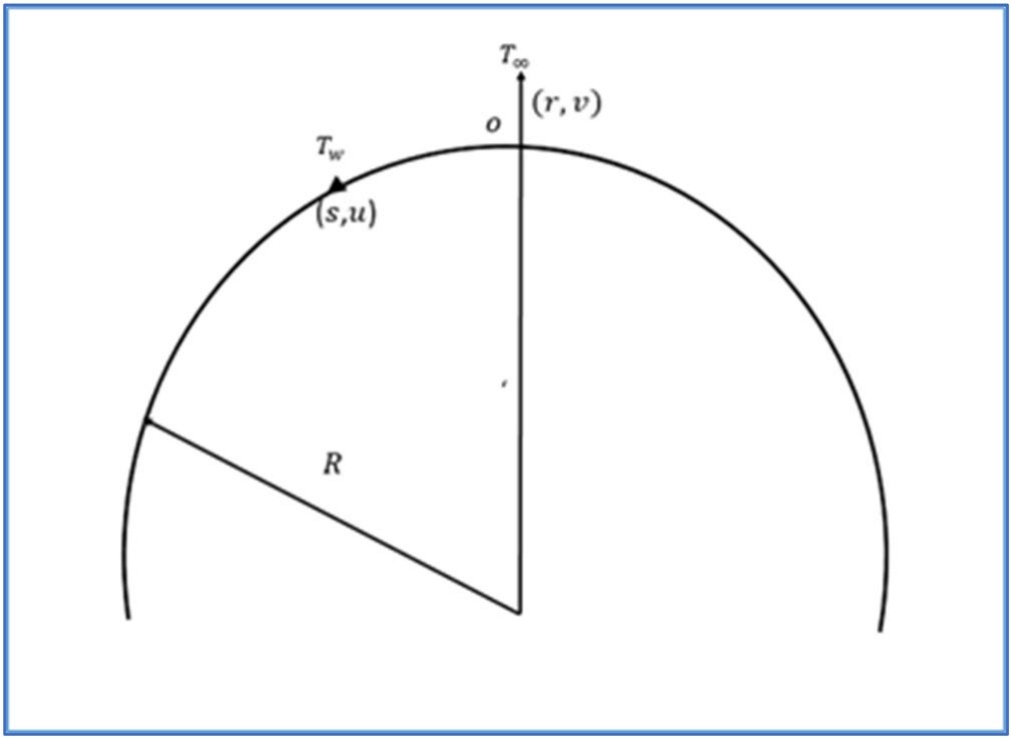
Flow geometry.

For this work, we consider steady, incompressible boundary driven flow of a micropolar fluid towards an exponentially curved stretched surface with subject to viscous dissipation. It is assumed that the sheet is stretching with exponential velocity of the form ${u}_{w}\left(s\right)=c{e}^\frac{s}{L},$ where $c$ is constant, having the dimension of velocity and $L$ represents the characteristic length. The surface has radius of curvature *R*. The schematic flow geometry is illustrated in Fig. 1. It is assumed the sheet has exponential temperature ${T}_{w}\left(s\right)={T}_{\infty }+{T}_{o}{e}^\frac{s}{L},$ where ${T}_{\infty }$ is ambient temperature and ${T}_{o}$ is constant. under the usual assumption the governing equation of the model are (see Sajid et al.^27^).

Mass conservation equation:

1 $${\left[\left(r+R\right)v\right]}_{r}+R{u}_{s}=0,$$

Momentum equation:

2 $$\frac{{u}^{2}}{r+R}=\frac{1}{\rho }{p}_{r},$$

3 $$v{u}_{r}+\frac{Ru}{r+R}{u}_{s}+\frac{uv}{r+R}=-\frac{1}{\rho }\frac{R}{r+R}{p}_{s}+\left(\nu +\frac{{k}^{*}}{\rho }\right)\left[{u}_{rr}+\frac{1}{r+R}{u}_{r}-\frac{u}{{\left(r+R\right)}^{2}} \right]-\frac{{k}^{*}}{\rho }{N}_{r}-\frac{\sigma {B}_{o}^{2}u}{\rho },$$

Angular Momentum equation:

4 $$v{N}_{r}+\frac{Ru}{r+R}{N}_{s}=\frac{{\gamma }^{*}}{\rho j}\left({N}_{rr}+\frac{1}{r+R}{N}_{r}\right)-\frac{{k}^{*}}{\rho j}\left(2N+{u}_{r}+\frac{u}{r+R}\right),$$

Energy equation:

5 $$\rho {c}_{p}\left(v{T}_{r}+\frac{Ru}{r+R}{T}_{s}\right)={k}_{O}\left({T}_{rr}+\frac{1}{r+R}{T}_{r}\right)+(\mu +{k}^{*}){\left({u}_{r}-\frac{u}{r+R}\right)}^{2},$$

*N* is the microrotation velocity, $\rho ,$ ${k}_{o},$ and ${c}_{P}$ are the density, thermal conductivity and specific heat respectively.

Here

6 $${\gamma }^{*} =\left(\mu +\frac{{k}^{*}}{2}\right)=\mu \left(1+\frac{{K}_{1}}{2}\right)j,$$

where ${K}_{1}=\frac{{k}^{*}}{\mu }\mathrm{ r}$epresents the material parameter, and $j=\frac{2\nu L}{c{e}^\frac{s}{L}}$ denotes micro inertial per nit mass, ${\gamma }^{*}$ and ${k}^{*}$ indicate the spin gradient and vortex viscosity respectively.

The boundary conditions for the problem are (see Okechi et al.^33^)

7 $$u={u}_{w}=c{e}^\frac{s}{L}, \;\; v=0, \;\; N=-{m}_{o}\frac{\partial u}{\partial r}, \;\;T={T}_{w} \, at \, r=0,$$

$$u\to 0, \;\; \frac{\partial u}{\partial r}\to 0, \;\; N\to 0, \;\; T\to {T}_{\infty } \, as \, r\to \infty$$

We define the following dimensionless variable transformations

8 $$u=c{e}^\frac{s}{L}f {^{\prime}}\left(\xi \right), v= -\frac{R}{r+R}\sqrt{\frac{c\nu {e}^\frac{s}{L}}{2L} } [f\left(\xi \right)+\xi f {^{\prime}}\left(\xi \right)],$$

9 $$\xi =\sqrt{\frac{c{e}^\frac{s}{L}}{2\nu L} }r, \;\; p=\rho {c}^{2}{e}^\frac{2s}{L}P\left(\xi \right),$$

10 $$N=c{e}^\frac{s}{L}\sqrt{\frac{c{e}^\frac{s}{L}}{2\nu L} }g\left(\xi \right), \;\; \theta =\frac{T-{T}_{\infty }}{{T}_{w}-{T}_{\infty }} .$$

By applying the transformation of Eqs. (8)–(10) on the system of Eqs. (1)–(5) the mass conversation is satisfied automatically and the remaining the Eqs. (2)–(5) reduced into the following form

11 $$\frac{{f}{{^{\prime}}2}}{\xi +\delta }={P}{^{\prime}},$$

12 $$\frac{4\delta }{\xi +\delta }P+\frac{\delta \xi }{\xi +\delta }{P}^{{\prime}}=\left(1+{K}_{1}\right)\left(f {^{\prime}}{^{\prime}}{^{\prime}}+\frac{{f}^{{^{\prime}}{^{\prime}}}}{\xi +\delta }-\frac{{f}^{{\prime}}}{{\left(\xi +\delta \right)}^{2}}\right)+\frac{\delta }{{\left(\xi +\delta \right)}^{2}}f{f}^{{\prime}}+\frac{\delta }{\xi +\delta }f{f}{{^{\prime}}{^{\prime}}}-\frac{(\xi +2\delta )}{{\left(\xi +\delta \right)}^{2}}\delta {{f}^{{\prime}}}^{2} -{K}_{1}{g}^{{\prime}}-{M}^{2}f{^{\prime}}$$

13 $$\left(1+\frac{{K}_{1}}{2}\right) \left({g}{{^{\prime}}{^{\prime}}}+\frac{{g}^{{\prime}}}{\xi +\delta }\right)-{K}_{1}\left(2g+{f}^{{{\prime}}{^{\prime}}}+\frac{{f}{^{\prime}}}{\xi +\delta }\right)+\frac{\delta }{\xi +\delta }f{g}^{{\prime}}-\frac{3\delta }{\xi +\delta }{f}^{{\prime}}g=0,$$

14 $${\theta }^{{{\prime}}{^{\prime}}}+\frac{{\theta }^{{\prime}}}{\xi +\delta }+\frac{\delta Pr}{\xi +\delta }\left(f{\theta }^{{\prime}}-2f {^{\prime}}\theta \right)+PrEc\left(1+{K}_{1} \right){\left({f}{{^{\prime}} {^{\prime}}}-\frac{{f}^{{\prime}}}{\xi +\delta }\right)}^{2}=0,$$

The pressure is eliminated from Eqs. (11)–(12)

15 $$(1+{K}_{1})\left({f}^{iv}+\frac{2{f}{{^{\prime}}{^{\prime}}{^{\prime}}}}{\upxi +\updelta }-\frac{{f}^{{{\prime}}{^{\prime}}}}{{\left(\upxi +\updelta \right)}^{2}}+\frac{{f}{{^{\prime}}}}{{\left(\upxi +\updelta \right)}^{3}}\right)+\frac{\delta }{\upxi +\updelta }f{f}{{^{\prime}} {^{\prime}}{^{\prime}}}+\frac{\delta }{{\left(\upxi +\updelta \right)}^{2}}f{f}^{{{\prime}}{^{\prime}}}-\frac{\delta }{{\left(\upxi +\updelta \right)}^{3}}f{f}^{{{\prime}}}-\frac{3\updelta }{\upxi +\updelta }{f}^{{{\prime}}}{f}^{{{\prime}} {^{\prime}}}-\frac{3\delta }{{\left(\upxi +\updelta \right)}^{2}}{{f} {{^{\prime}}}}^{2}-{K}_{1}\left({g}{{^{\prime}} {^{\prime}}}+\frac{{g}^{{\prime}}}{\xi +\delta }\right)-{M}^{2}\left({f}{{^{\prime}}{^{\prime}}}+\frac{{f}^{{\prime}}}{\xi +\delta }\right)=0.$$

${M}^{2}= \frac{2\sigma {B}_{o}^{2}L }{\rho c}$ is the magnetic parameter, $\delta =\sqrt{\frac{c{e}^\frac{s}{L}}{2\nu L}}R$ is the radius of curvature, $Pr= \frac{{k}_{o}}{\mu cp}$ and $Ec= \frac{{u}_{w}^{2}}{{c}_{p}({T}_{w}-{T}_{\infty })}$ denote Prandtl and Eckert number, respectively.

The boundary conditions become

16 $$f\left(0\right)=0, \;\; {f}^{{\prime}}\left(0\right)=1, \;\; g\left(0\right)=-{m}_{0}{f} {{^{\prime}} {^{\prime}}}\left(0\right), \;\; \theta \left(0\right)=1 \; at \; \xi =0,$$

17 $${f}^{{\prime}}\left(\xi \right)=0, \;\; {f}{{^{\prime}}{^{\prime}}}(\xi )=0, \;\; g\left(\xi \right)=0, \;\; \theta \left(\xi \right)=0 as \xi \; \to \; \infty .$$

where ${m}_{0}$ (0 $\le {m}_{0}\le 1$) is a constant.

The skin friction coefficient ${C}_{fs}$ for our physical model is given as

18 $${C}_{fs}=\frac{{\tau }_{rs}}{\rho {u}_{w}^{2}}, \;\; {\tau }_{rs}={\left[\left(\mu +{k}^{*}\right)\left(\frac{\partial u}{\partial r}-\frac{u}{r+R}\right)+\delta N \right]}_{r=0},$$

where ${\tau }_{rs}$ imply the wall shear stress.

The dimensionless expression for skin friction is

19 $$\sqrt{\frac{L}{2s}} {Re}_{s}^\frac{1}{2}{C}_{fs}=\left(1+{K}_{1}\right)\left({f}{{^{\prime}}{^{\prime}}}\left(0\right)-\frac{{f}^{{\prime}}\left(0\right)}{\delta }\right),$$

For temperature distribution the local heat transfer rate is given as

20 $$N{u}_{s}=\frac{s{q}_{w}}{{k}_{o}\left({T}_{w}-{T}_{\infty }\right)}, \;\; q_{w}= -{k}_{o}{\left(\frac{\partial T}{\partial r}\right)}_{r=0},$$

where ${q}_{w}$ represents the wall heat flux.

It can be written in non-dimensional form as follow

21 $$\sqrt{\frac{2L}{s}} {Re}_{s}^{-\frac{1}{2}}N{u}_{s}= -{\theta }^{{\prime}}\left(0\right).$$

Couple stress on the surface is define as

22 $${C}_{s}=\frac{{M}_{w}}{\mu j{U}_{w}}, \;\;\;{M}_{w}=\gamma {\left(\frac{\partial N}{\partial r}\right)}_{r=0},$$

23 $$2Ls{{Re}_{s}^{-1}C}_{s}=\left(1+\frac{{K}_{1}}{2}\right)g{^{\prime}}(0)$$

The local Reynolds number is ${Re}_{s}=\frac{{u}_{w}s}{\nu }.$

## Numerical scheme

The non-linear ODEs Eqs. (13)–(15) with boundary conditions (16)–(17) are solved numerically by applying two independent numerical methods; (1) Keller box method and (2) shooting method.

We define new variables for the implementations of these numerical techniques,

24 $${f}^{{\prime}}=u, \;\; {f}{{^{\prime}}{^{\prime}}}=v\;\; {f}{{^{\prime}}{^{\prime}} {^{\prime}}}=w, \;\; { g}^{{\prime}}=p, { \theta }^{{\prime}}=q,$$

25 $${w}^{{\prime}}+{A}_{1}w+{A}_{2}v+{A}_{3}u+{A}_{4}fw+{A}_{5}fv+{A}_{6}fu+{A}_{7}{u}^{2}+{A}_{8}uv+{A}_{9}{p}^{{\prime}}+{A}_{10}p=0$$

26 $${p}^{{\prime}}+{B}_{1}p+{B}_{2}g+{B}_{3}v+{B}_{4}u+{B}_{5}fp+{B}_{6}ug=0,$$

27 $${q}^{{\prime}}+{B}_{1}q+{B}_{7}fq+{B}_{8}u\theta +{B}_{9}{v}^{2}+{B}_{10}uv+{B}_{11}{u}^{2}=0,$$

where $${A}_{1}=\frac{2}{\xi +\delta },$$ $${A}_{2}=-\frac{1}{{\left(\xi +\delta \right)}^{2}}-\frac{{M}^{2}}{1+{K}_{1}},$$ $${A}_{3}=\frac{1}{{\left(\xi +\delta \right)}^{3}}-\frac{{M}^{2}}{\left(\xi +\delta \right)\left(1+{K}_{1}\right)},$$ $${A}_{4}=\frac{\delta }{\left(\xi +\delta \right)(1+{K}_{1})},$$ $${A}_{5}=\frac{\delta }{{\left(\xi +\delta \right)}^{2}(1+{K}_{1})},$$ $${A}_{6}=-\frac{\delta }{{\left(\xi +\delta \right)}^{3}(1+{K}_{1})},$$ $${A}_{7}= -\frac{3\delta }{{\left(\xi +\delta \right)}^{2}(1+{K}_{1})},$$ $${A}_{8}=-\frac{3\delta }{\left(\xi +\delta \right)\left(1+{K}_{1}\right)},$$ $${A}_{9}=-\frac{{K}_{1}}{1+{K}_{1}},$$ $${A}_{10}=-\frac{{K}_{1}}{(\xi +\delta )(1+{K}_{1})},$$ $${B}_{1}=\frac{1}{(\xi +\delta )},$$ $${B}_{2}=-\frac{2{K}_{1}}{(1+\frac{{K}_{1}}{2})},$$ $${B}_{3}=-\frac{{K}_{1}}{(1+\frac{{K}_{1}}{2})},$$ $${B}_{4}=-\frac{{K}_{1}}{(\xi +\delta )(1+\frac{{K}_{1}}{2})},$$ $${B}_{5}=\frac{\delta }{(\xi +\delta )(1+\frac{{K}_{1}}{2})},$$ $${B}_{6}=-\frac{3\delta }{2(\xi +\delta )(1+\frac{{K}_{1}}{2})},$$ $${B}_{7}=\frac{\delta Pr}{\xi +\delta },$$ $${B}_{8}=-\frac{2\delta Pr}{\xi +\delta },$$ $${B}_{9}=PrEc,$$ $${B}_{10}= -\frac{2PrEc}{\xi +\delta },$$ $${B}_{11}=\frac{PrEC}{{\left(\xi +\delta \right)}^{2}}.$$

Boundary conditions (16) and (17) become

28 $$f\left(0\right)=0, \;\; u\left(0\right)=1, \;\; g\left(0\right)=-{m}_{o}v\left(o\right), \;\; \theta \left(0\right)=1,$$

29 $$u\left(\infty \right)=0, \;\; v\left(\infty \right)=0, \;\; g\left(\infty \right)=0,\;\; \theta \left(\infty \right)=0.$$

The transformed problem given by Eqs. (24)–(27) subject to boundary condition (28) and (29) are solved numerically via implicit finite difference scheme referred to as Keller box method (see Cebeci and Bradshaw^37^).

Net points are defined as

30 $${\xi }_{1}=0, {\xi }_{j}={\xi }_{j-1}+{h}_{j}, \quad j=2, 3,\dots ,J, \;\; {\xi }_{J}={\xi }_{\infty }.$$

Using center finite difference approximation at the mid-point of ${\xi }_{j-\frac{1}{2}}$ in Eqs. (24)–(27)

31 $${h}_{j}^{-1}\left({f}_{j}^{i}-{f}_{j-1}^{i}\right)={u}_{j-\frac{1}{2}}^{i},$$

32 $${h}_{j}^{-1}\left({u}_{j}^{i}-{u}_{j-1}^{i}\right)={v}_{j-\frac{1}{2}}^{i},$$

33 $${h}_{j}^{-1}\left({v}_{j}^{i}-{v}_{j-1}^{i}\right)={w}_{j-\frac{1}{2}}^{i},$$

34 $${h}_{j}^{-1}\left({w}_{j}^{i}-{w}_{j-1}^{i}\right)+{A}_{1}{w}_{j-\frac{1}{2}}^{i}+{A}_{2}{v}_{j-\frac{1}{2}}^{i}+{A}_{3}{u}_{j-\frac{1}{2}}^{i}+{A}_{4}(f{w)}_{j-\frac{1}{2}}^{i}+{A}_{5}(f{v)}_{j-\frac{1}{2}}^{i}+{A}_{6}(f{u)}_{j-\frac{1}{2}}^{i}+{A}_{7}{\left({u}^{2}\right)}_{j-\frac{1}{2}}^{i}+{A}_{8}(uv{)}_{j-\frac{1}{2}}^{i}+{A}_{9}\left({p}_{j}^{i}-{p}_{j-1}^{i}\right){h}_{j}^{-1}+{A}_{10}{p}_{j-\frac{1}{2}}^{i}=0$$

35 $${h}_{j}^{-1}\left({g}_{j}^{i}-{g}_{j-1}^{i}\right)={p}_{j-\frac{1}{2}}^{i},$$

36 $${h}_{j}^{-1}\left({p}_{j}^{i}-{p}_{j-1}^{i}\right)+{B}_{1}{p}_{j-\frac{1}{2}}^{i}+{B}_{2}\left(2{g}_{j-\frac{1}{2}}^{i}+{v}_{j-\frac{1}{2}}^{i}\right)+{B}_{3}{u}_{j-\frac{1}{2}}^{i}+{B}_{4}(f{p)}_{j-\frac{1}{2}}^{i}+{B}_{5}(f{v)}_{j-\frac{1}{2}}^{i}=0,$$

37 $${h}_{j}^{-1}\left({\theta }_{j}^{i}-{\theta }_{j-1}^{i}\right)={q}_{j-\frac{1}{2}}^{i},$$

38 $${{h}_{j}^{-1}\left({q}_{j}^{i}-{q}_{j-1}^{i}\right)+B}_{1}{q}_{j-\frac{1}{2}}^{i}+{B}_{6}(f{q)}_{j-\frac{1}{2}}^{i}+{B}_{7}(u{\theta )}_{j-\frac{1}{2}}^{i}+{B}_{8}(v{)}_{j-\frac{1}{2}}^{i}+{B}_{9}{u}_{j-\frac{1}{2}}^{i}=0,$$

The corresponding boundary conditions are

39 $${f}_{1}^{i}=0, { u}_{1}^{i}=1, {g}_{1}^{i}=-{m}_{0}v\left(0\right), { \theta }_{1}^{i}=1, { v}_{J}^{i}=0, { w}_{J}^{i}=0,{ g}_{J}^{i}=0, {\theta }_{J}^{i}=0.$$

Equations (31)–(38) are a system of nonlinear equation, Newton’s quasi-linearization approach is used to solve these equations, thus we have

$${f}_{1}^{i}=0, { u}_{1}^{i}=1, {g}_{1}^{i}=-{m}_{0}v\left(0\right), { \theta }_{1}^{i}=1, { v}_{J}^{i}=0, { w}_{J}^{i}=0,{ g}_{J}^{i}=0, {\theta }_{J}^{i}=0$$

40 $${f}_{j}^{(k+1)}={f}_{j}^{(k)}+\delta {f}_{j}^{(k)}, { u}_{j}^{(k+1)}={u}_{j}^{(k)}+\delta {u}_{j}^{(k)},{ v}_{j}^{(k+1)}={v}_{j}^{(k)}+\delta {v}_{j}^{(k+1) }, {{w}_{j}^{(k+1)}={w}_{j}^{(k)}+\delta {w}_{j}^{(k) }, g}_{j}^{(k+1)}={g}_{j}^{(k)}+\delta {g}_{j}^{(k)}, { p}_{j}^{(k+1)}={p}_{j}^{(k)}+\delta {p}_{j}^{(k)}, {{\theta }_{j}^{(k+1)}={\theta }_{j}^{(k)}+\delta {\theta }_{j}^{(k)}, q}_{j}^{(k+1)}={q}_{j}^{(k)}+\delta {q}_{j}^{(k) }.$$

Using Eq. (40) into Eqs. (31)–(38) and ignoring the quadratic terms of $\delta$

41 $$\delta {f}_{j}-\delta {f}_{j-1}-\frac{1}{2}{h}_{j}\left(\delta {u}_{j}+\delta {u}_{j-1}\right)={\left({r}_{1}\right)}_{j-\frac{1}{2}},$$

42 $$\delta {u}_{j}-\delta {u}_{j-1}-\frac{1}{2}{h}_{j}\left(\delta {v}_{j}+\delta {v}_{j-1}\right)={\left({r}_{2}\right)}_{j-\frac{1}{2}},$$

43 $$\delta {v}_{j}-\delta {v}_{j-1}-\frac{1}{2}{h}_{j}\left(\delta {w}_{j}+\delta {w}_{j-1}\right)={\left({r}_{3}\right)}_{j-\frac{1}{2}},$$

44 $${\left({\alpha }_{1}\right)}_{j}\delta {v}_{j}+{\left({\alpha }_{2}\right)}_{j}\delta {v}_{j-1}+{\left({\alpha }_{3}\right)}_{j}\delta {f}_{j+}{\left({\alpha }_{4}\right)}_{j}\delta {f}_{j-1}+{\left({\alpha }_{5}\right)}_{j}\delta {u}_{j}+{\left({\alpha }_{6}\right)}_{j}\delta {u}_{j-1}+{\left({\alpha }_{7}\right)}_{j}\delta {w}_{j}+{\left({\alpha }_{8}\right)}_{j}\delta {w}_{j-1}+{\left({\alpha }_{9}\right)}_{j}\delta {p}_{j}+{\left({\alpha }_{10}\right)}_{j}\delta {p}_{j-1}={\left({r}_{4}\right)}_{j-\frac{1}{2}},$$

45 $$\delta {g}_{j}-\delta {g}_{j-1}-\frac{1}{2}{h}_{j}\left(\delta {p}_{j}+\delta {p}_{j-1}\right)={\left({r}_{5}\right)}_{j-\frac{1}{2}},$$

46 $${\left({\alpha }_{11}\right)}_{j}\delta {f}_{j}+{\left({\alpha }_{12}\right)}_{j}\delta {f}_{j-1}+{\left({\alpha }_{13}\right)}_{j}\delta {u}_{j+}{\left({\alpha }_{14}\right)}_{j}\delta {u}_{j-1}+{\left({\alpha }_{15}\right)}_{j}\delta {g}_{j}+{\left({\alpha }_{16}\right)}_{j}\delta {g}_{j-1}+{\left({\alpha }_{17}\right)}_{j}\delta {v}_{j}+{\left({\alpha }_{18}\right)}_{j}\delta {v}_{j-1}+{\left({\alpha }_{19}\right)}_{j}\delta {p}_{j}+{\left({\alpha }_{20}\right)}_{j}\delta {p}_{j-1}={\left({r}_{6}\right)}_{j-\frac{1}{2}},$$

47 $$\delta {\theta }_{j}-\delta {\theta }_{j-1}-\frac{1}{2}{h}_{j}\left(\delta {q}_{j}+\delta {q}_{j-1}\right)={\left({r}_{7}\right)}_{j-\frac{1}{2}},$$

48 $${\left({\alpha }_{21}\right)}_{j}\delta {f}_{j}+{\left({\alpha }_{22}\right)}_{j}\delta {f}_{j-1}+{\left({\alpha }_{23}\right)}_{j}\delta {u}_{j+}{\left({\alpha }_{24}\right)}_{j}\delta {u}_{j-1}+{\left({\alpha }_{25}\right)}_{j}\delta {\theta }_{j}+{\left({\alpha }_{26}\right)}_{j}\delta {\theta }_{j-1}+{\left({\alpha }_{27}\right)}_{j}\delta {v}_{j}+{\left({\alpha }_{28}\right)}_{j}\delta {v}_{j-1}+{\left({\alpha }_{29}\right)}_{j}\delta {q}_{j}+{\left({\alpha }_{30}\right)}_{j}\delta {q}_{j-1}={\left({r}_{8}\right)}_{j-\frac{1}{2}}.$$

Boundary conditions after applying the Newton’s quasi-linearization approach become

49 $$\delta {f}_{1}=0, \delta {u}_{1}=0, \delta {g}_{1}=0, { \delta \theta }_{1}=0, { \delta v}_{J}=0, { \delta w}_{J}=0,{ \delta g}_{J}=0, { \delta \theta }_{J}=0,$$

where

50 $${\left({r}_{1}\right)}_{j-\frac{1}{2}}={f}_{j-1}-{f}_{j}+{h}_{j}{u}_{j-\frac{1}{2}},$$

51 $${\left({r}_{2}\right)}_{j-\frac{1}{2}}={u}_{j-1}-{u}_{j}+{h}_{j}{v}_{j-\frac{1}{2}},$$

52 $${\left({r}_{3}\right)}_{j-\frac{1}{2}}={v}_{j-1}-{v}_{j}+{h}_{j}{w}_{j-\frac{1}{2}},$$

53 $${\left({r}_{4}\right)}_{j-\frac{1}{2}}=-\left\{{{(w}_{j}-{w}_{j-1}){h}_{j}^{-1}+A}_{1}{w}_{j-\frac{1}{2}}+{A}_{2}{v}_{j-\frac{1}{2}}+{A}_{3}{u}_{j-\frac{1}{2}}+{A}_{4}(f{w)}_{j-\frac{1}{2}}+{A}_{5}(f{v)}_{j-\frac{1}{2}}{+A}_{6}(f{u)}_{j-\frac{1}{2}}+{A}_{7}{u}_{j-\frac{1}{2}}^{2}+{A}_{8}(uv{)}_{j-\frac{1}{2}}+{A}_{9}\left({p}_{j}-{p}_{j-1}\right){h}_{j}+{A}_{10}{p}_{j-\frac{1}{2}}\right\},$$

54 $${\left({r}_{5}\right)}_{j-\frac{1}{2}}={g}_{j-1}-{g}_{j}+{h}_{j}{p}_{j-\frac{1}{2}},$$

55 $${\left({r}_{6}\right)}_{j-\frac{1}{2}}=-\left\{{\left({p}_{j}-{p}_{j-1 }\right){h}_{j}+{B}_{1}p}_{j-\frac{1}{2}}+{B}_{2}{g}_{j-\frac{1}{2}}+{B}_{3}{v}_{j-\frac{1}{2}}+{B}_{4}{u}_{j-\frac{1}{2}}+{B}_{5}(fp{)}_{j-\frac{1}{2}}+{B}_{6}(ug{)}_{j-\frac{1}{2}} \right\},$$

56 $${\left({r}_{7}\right)}_{j-\frac{1}{2}}={\theta }_{j-1}-{\theta }_{j}+{h}_{j}{q}_{j-\frac{1}{2}},$$

57 $${\left({r}_{8}\right)}_{j-\frac{1}{2}}=-\left\{{({q}_{j}-{q}_{j-1 }){h}_{j}+B}_{1}{q}_{j-\frac{1}{2}}+{B}_{7}(f{q)}_{j-\frac{1}{2}}+{B}_{8}(u\theta {)}_{j-\frac{1}{2}}+{B}_{9}{v}_{j-\frac{1}{2}}^{2}+{B}_{10}u{v}_{j-\frac{1}{2}}+{B}_{11}{u}_{j-\frac{1}{2}}^{2}\right\},$$

Coefficients are

- $${\left({\alpha }_{1}\right)}_{j}={\left({\alpha }_{2}\right)}_{j}=\frac{{A}_{2}}{2}+\frac{{A}_{5}}{2}{f}_{j-\frac{1}{2}}+\frac{{A}_{8}}{2}{u}_{j-\frac{1}{2}},$$
- $${\left({\alpha }_{3}\right)}_{j}={\left({\alpha }_{4}\right)}_{j}=\frac{{A}_{4}}{2}{w}_{j-\frac{1}{2}}+\frac{{A}_{5}}{2}{v}_{j-\frac{1}{2}}+\frac{{A}_{6}}{2}{u}_{j-\frac{1}{2}},$$
- $${\left({\alpha }_{5}\right)}_{j}={\left({\alpha }_{6}\right)}_{j}=\frac{{A}_{3}}{2}+\frac{{A}_{6}}{2}{f}_{j-\frac{1}{2}}+{A}_{7}{u}_{j-\frac{1}{2}}+\frac{{A}_{8}}{2}{v}_{j-\frac{1}{2}},$$
- $${\left({\alpha }_{7}\right)}_{j}=\frac{1}{{h}_{j}}+\frac{1}{2}{A}_{1}+\frac{1}{2}{A}_{4}{f}_{j-\frac{1}{2}}$$,
- $${\left({\alpha }_{8}\right)}_{j}=-\frac{1}{{h}_{j}}+\frac{1}{2}{A}_{1}+\frac{1}{2}{A}_{4}{f}_{j-\frac{1}{2}},$$
- $${\left({\alpha }_{9}\right)}_{j}=\frac{{A}_{9}}{{h}_{j}}+\frac{{A}_{10}}{2} ,{\left({\alpha }_{10}\right)}_{j}=-\frac{{A}_{9}}{{h}_{j}}+\frac{{A}_{10}}{2},$$
- $${\left({\alpha }_{11}\right)}_{j}={\left({\alpha }_{12}\right)}_{j}=\frac{{B}_{5}}{2}{p}_{j-\frac{1}{2}},{\left({\alpha }_{13}\right)}_{j}={\left({\alpha }_{14}\right)}_{j}=\frac{{B}_{4}}{4}+\frac{{B}_{6}}{2}{g}_{j-\frac{1}{2}},$$
- $${\left({\alpha }_{15}\right)}_{j}={\left({\alpha }_{16}\right)}_{j}=\frac{{B}_{2}}{2}+\frac{{B}_{6}}{2}{u}_{j-\frac{1}{2}}, {\left({\alpha }_{17}\right)}_{j}={\left({\alpha }_{18}\right)}_{j}=\frac{{B}_{3}}{2},$$
- $${\left({\alpha }_{19}\right)}_{j}=\frac{1}{{h}_{j}}+\frac{{B}_{1}}{2}+\frac{{B}_{5}}{2}{f}_{j-\frac{1}{2}}{,\left({\alpha }_{20}\right)}_{j}=-\frac{1}{{h}_{j}}+\frac{{B}_{1}}{2}+\frac{{B}_{5}}{2}{f}_{j-\frac{1}{2}},$$
- $${\left({\alpha }_{21}\right)}_{j}={\left({\alpha }_{22}\right)}_{j}=\frac{{B}_{7}}{2}{q}_{j-\frac{1}{2}},{\left({\alpha }_{23}\right)}_{j}={\left({\alpha }_{24}\right)}_{j}=\frac{{B}_{8}}{2}{\theta }_{j-\frac{1}{2}}+\frac{{B}_{10}}{2}{v}_{j-\frac{1}{2}}+{B}_{11}{u}_{j-\frac{1}{2}},$$
- $${\left({\alpha }_{25}\right)}_{j}={\left({\alpha }_{26}\right)}_{j}=\frac{{B}_{8}}{2}{u}_{j-\frac{1}{2}},{\left({\alpha }_{27}\right)}_{j}={\left({\alpha }_{28}\right)}_{j}=\frac{{B}_{9}}{2}{v}_{j-\frac{1}{2} }+\frac{{B}_{10}}{2}{u}_{j-\frac{1}{2}}$$**,**
- $${\left({\alpha }_{29}\right)}_{j}=\frac{1}{{h}_{j}}+\frac{{B}_{1}}{2}+\frac{{B}_{7}}{2}{f}_{j-\frac{1}{2}},{\left({\alpha }_{30}\right)}_{j}=-\frac{1}{{h}_{j}}+\frac{{B}_{1}}{2}+\frac{{B}_{7}}{2}{f}_{j-\frac{1}{2}}$$**.**

Equations (51)–(57) are the system of linear algebraic equations. These systems of equations are solved by block tridiagonal elimination method.

To solve the system of ODEs Eqs. (24)–(27) with shooting method, an initial guess value must be needed, for the define new variables as $v\left(0\right)={d}_{1}, w\left(0\right)={d}_{2}, p\left(0\right)= {d}_{3}, q(0)={d}_{4}$ and the numerical solution can be attained using IVPs by Runge–Kutta method of order 6. If the condition given by^29^ are correct up to the given accuracy ${10}^{-6}$ then our procedure is correct otherwise we take another guess and perform the computation again.

## Results and discussion

**Figure 2:**
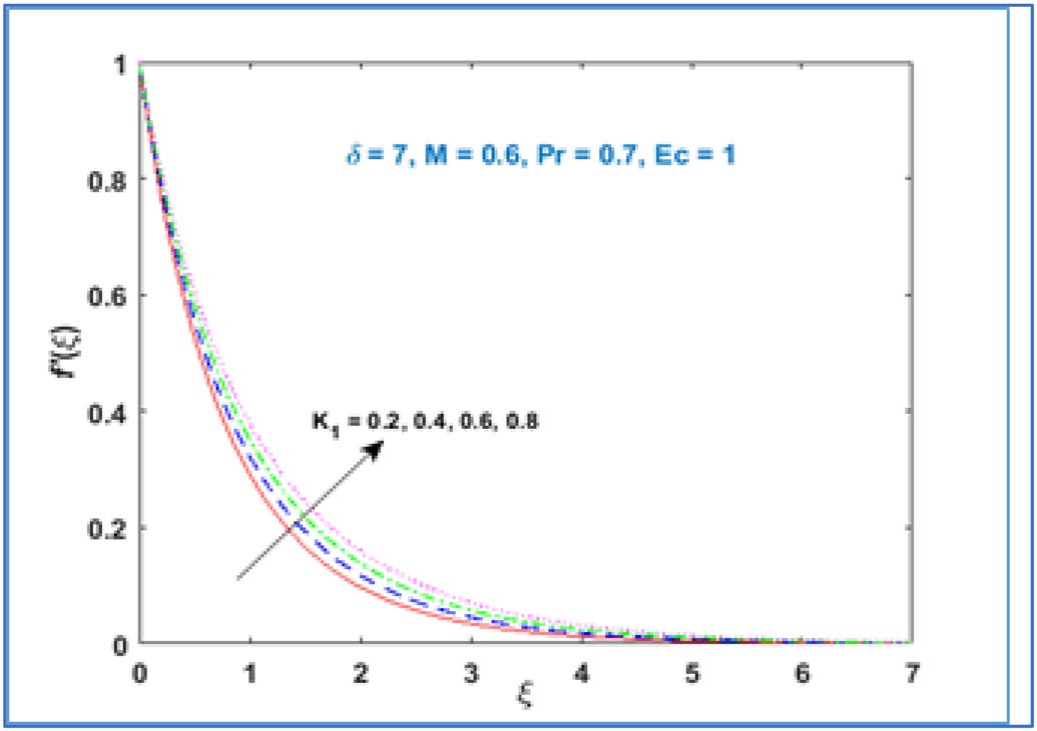
$${f}^{{\prime}}\left(\xi \right)\;\; versus \; \; {K}_{1}.$$

**Figure 3:**
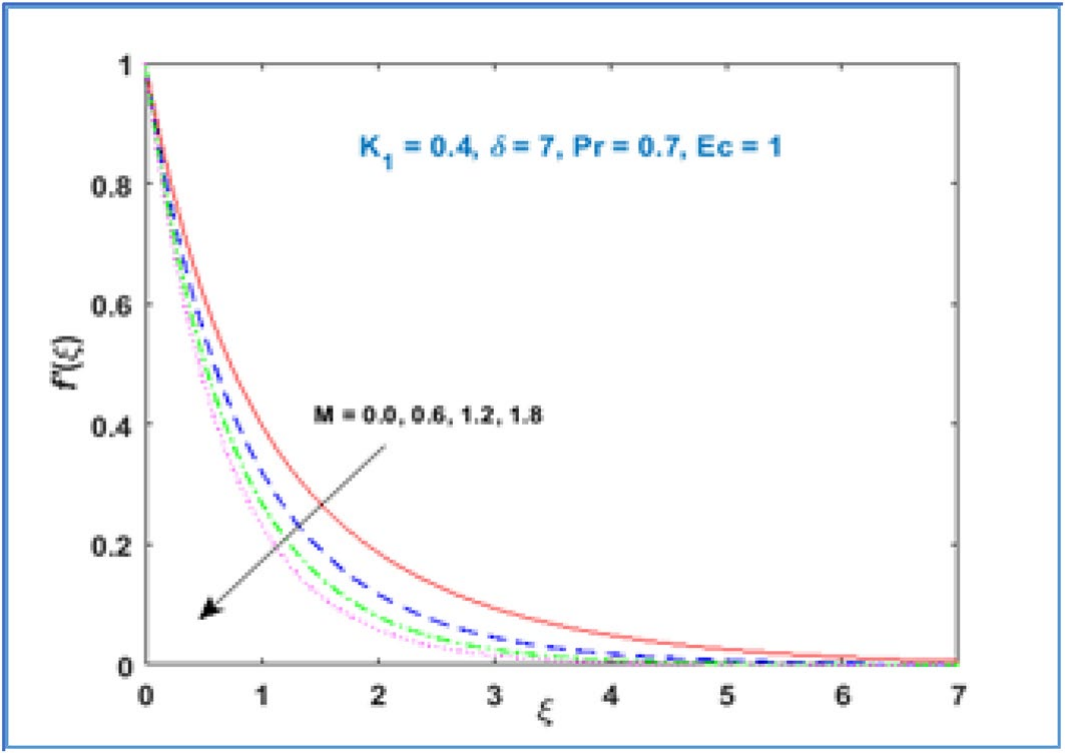
$${f}^{{\prime}}\left(\xi \right)\;\; versus \; \; M.$$

**Figure 4:**
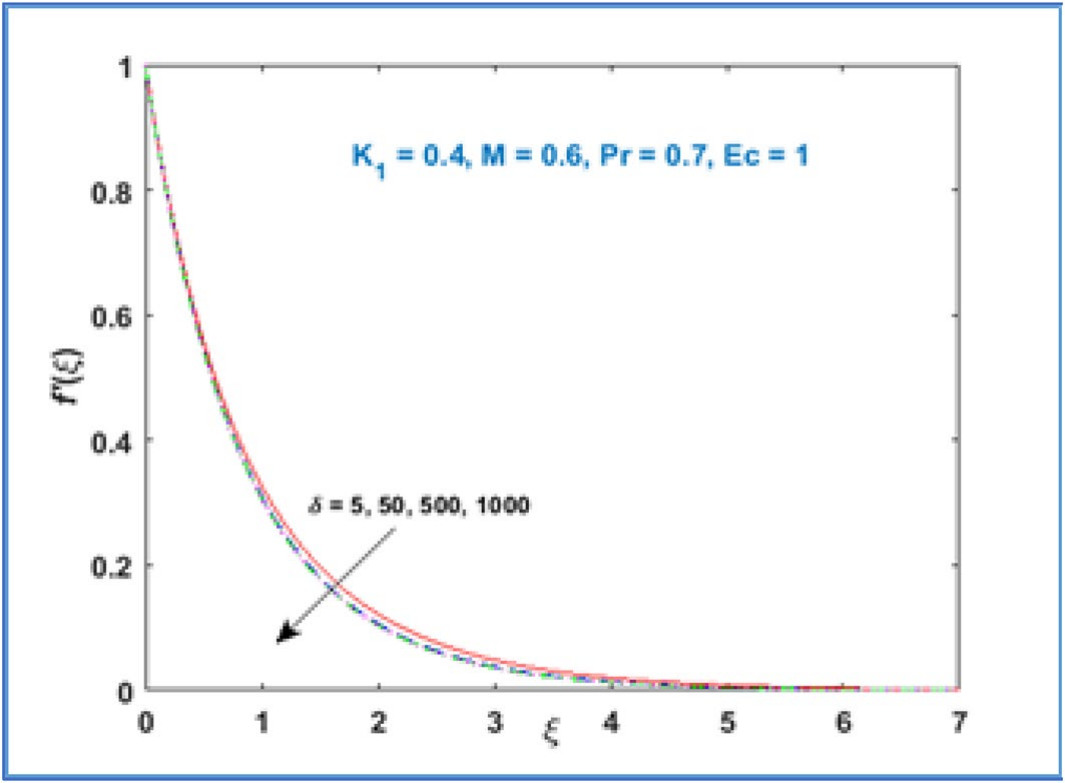
$${f}^{{\prime}}\left(\xi \right)\;\; versus \; \; \delta .$$

In Fig. 2 the variation of material parameter ${K}_{1}$ is shown on velocity profile $f{^{\prime}}(\xi )$. It has been noticed that velocity of fluid rises with growing values of material parameter ${K}_{1}.$ As we increase material parameter the micro concentration of the fluid increased which alter the flow and as a result the boundary layer thickness enhances. Figure 3 is sketched to know the behavior of magnetic parameter $M$ on velocity profile. This figure discloses that velocity profile declines for large estimation of magnetic parameter $M.$ The magnetic force is a resistive quantity which works against the flow, as a result the velocity decreases as shown in Fig. 3. Figure 4 interprets the effect of radius of curvature $\delta$ on the fluid velocity. As large the radius of curvature parameter $\delta$ values, velocity decreases.

**Figure 5:**
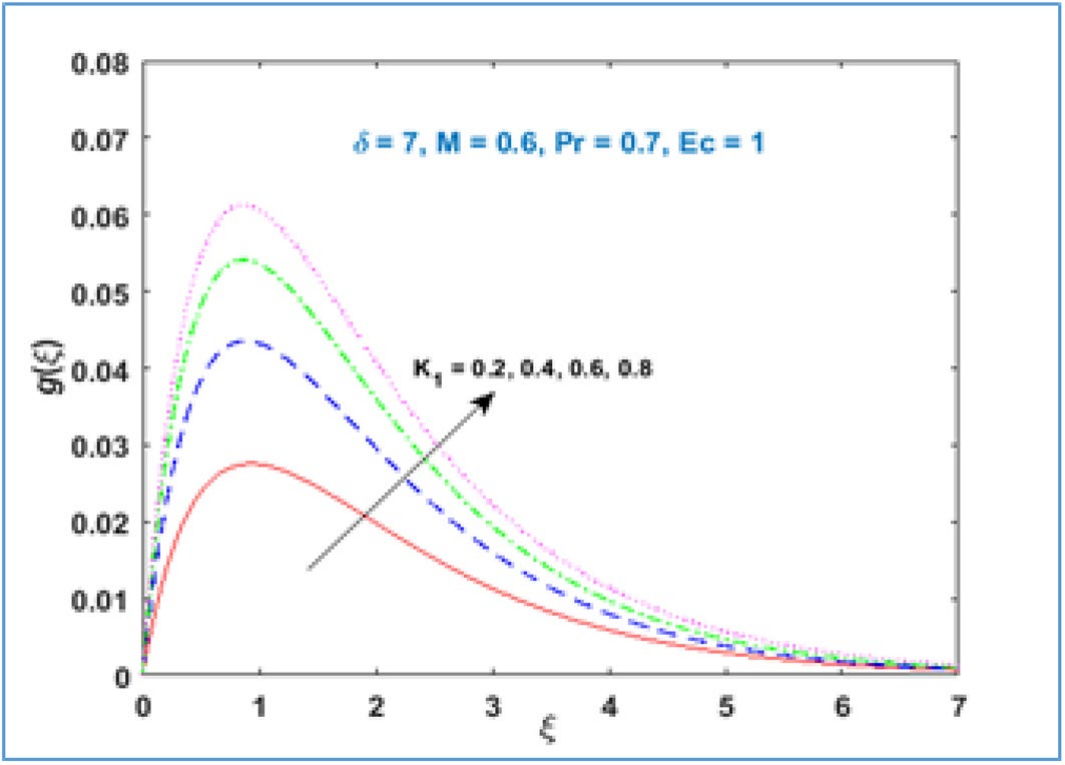
$$g\left(\xi \right)\;\; versus \; \; {K}_{1}.$$

**Figure 6:**
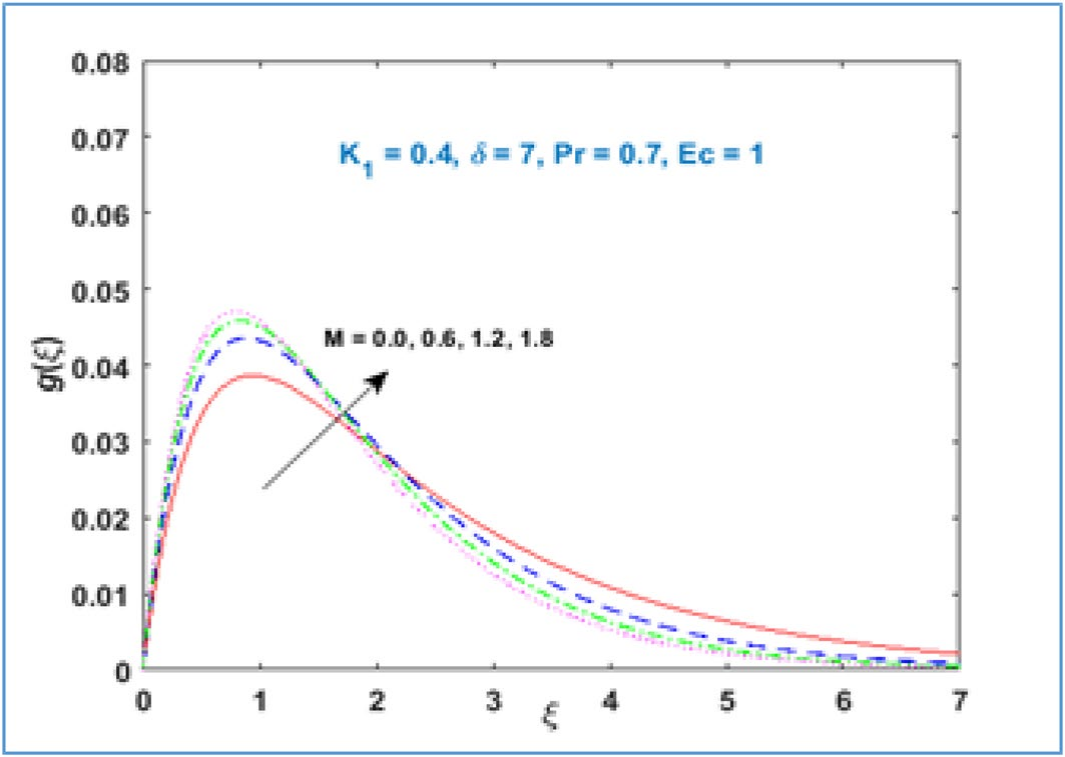
$$g\left(\xi \right)\;\; versus \; \; M.$$

**Figure 7:**
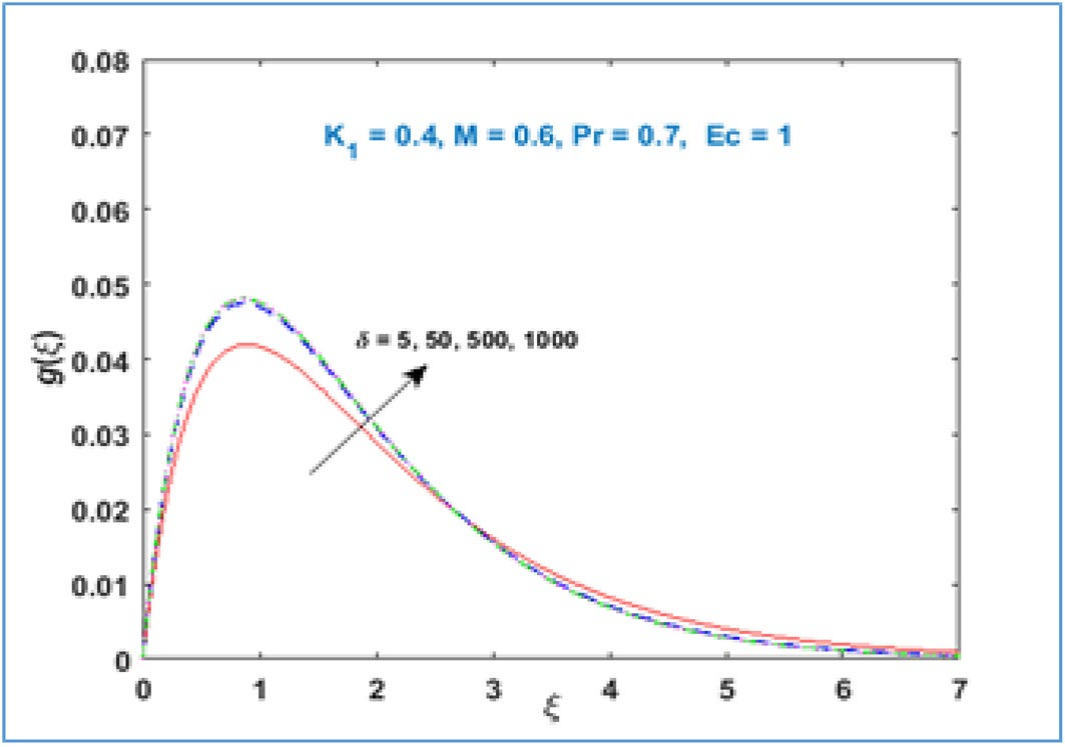
$$g\left(\xi \right)\;\; versus \; \; \delta .$$

Material parameter ${K}_{1}$ behavior is described through Fig. 5. It is inspected that microrotation velocity accelerates for large values of material parameter ${K}_{1}$. Figure 6 illustrates the behavior of microrotation profile with magnetic parameter $M$. It can be noticed that near the stretching surface the microrotation profile enhances, the profile overlaps far away from the sheet and then decreases as given in Fig. 6. Figure 7 represents the impact of radius of curvature $\delta$ parameter on the microrotation profile. The curvature parameter $\delta$ increases near the stretching sheet, opposite behavior is perceived as one moving farther from the stretching surface.

**Figure 8:**
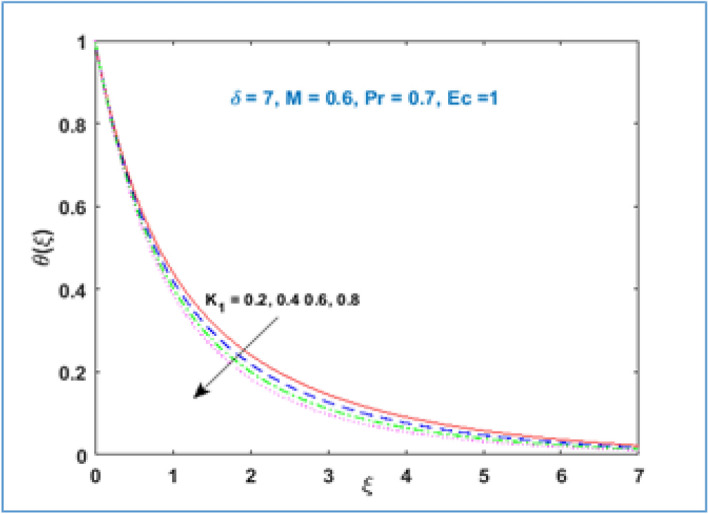
$$\theta \left(\xi \right)\;\; versus \; \; {K}_{1}.$$

**Figure 9:**
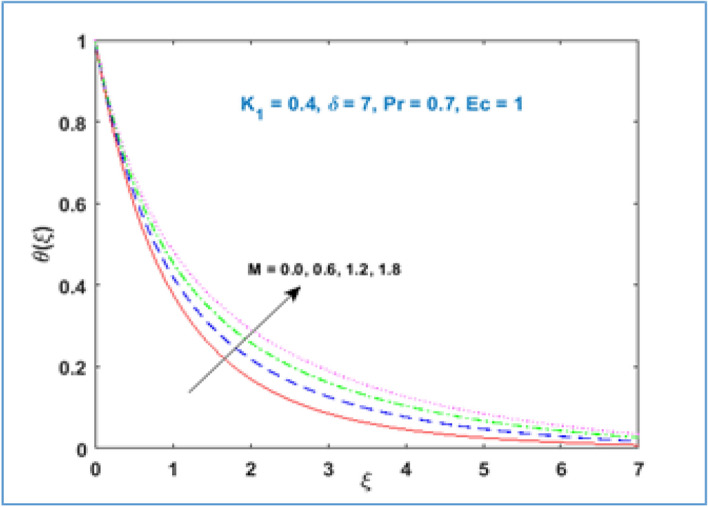
$$\theta \left(\xi \right)\;\; versus \; \; M.$$

**Figure 10:**
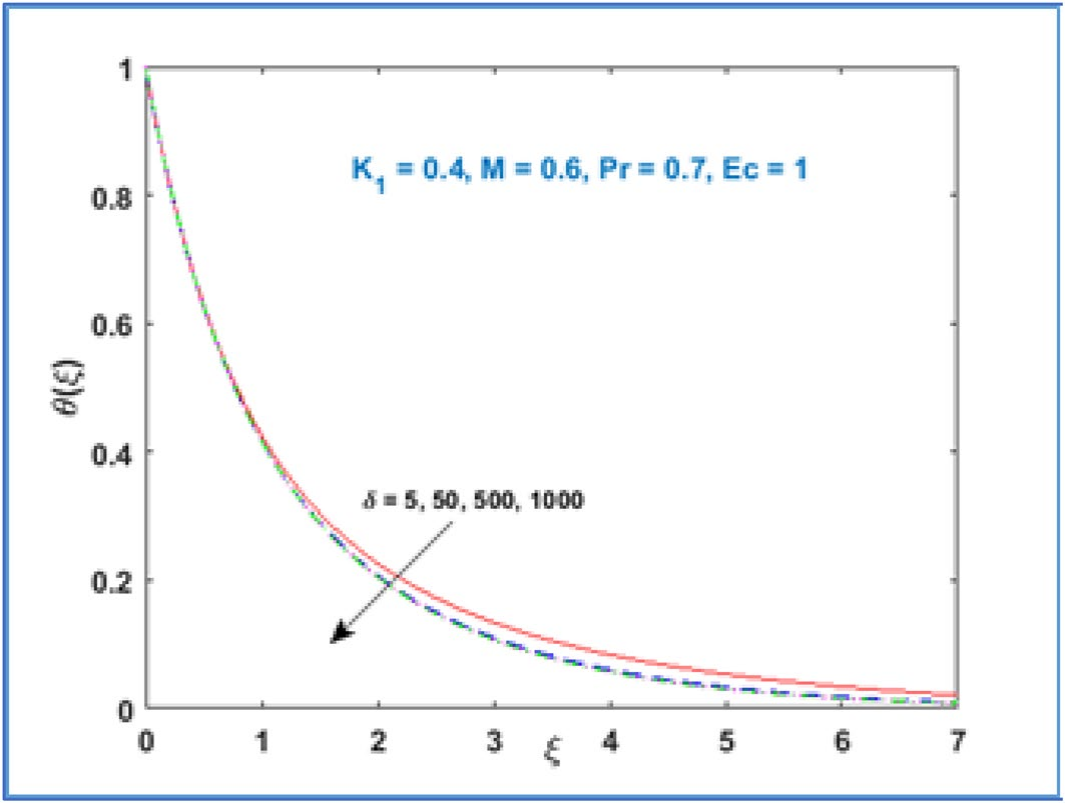
$$\theta \left(\xi \right)\;\; versus \; \; \delta .$$

**Figure 11:**
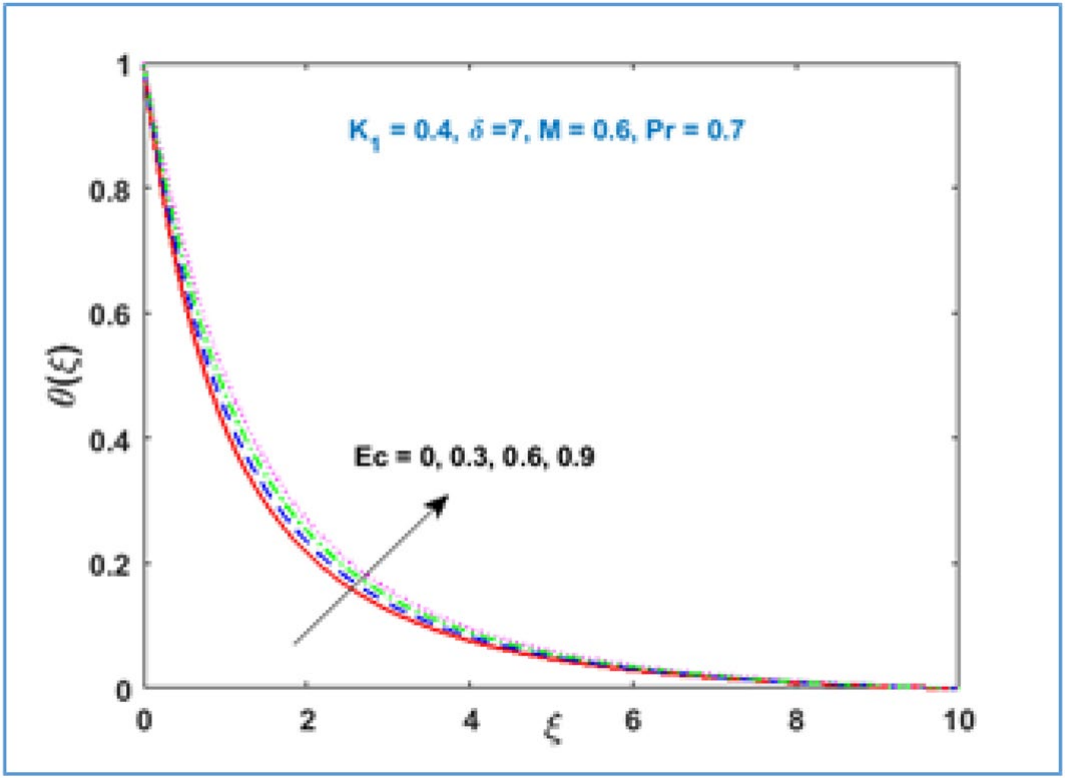
$$\theta \left(\xi \right)\;\; versus \; \; Ec.$$

**Figure 12:**
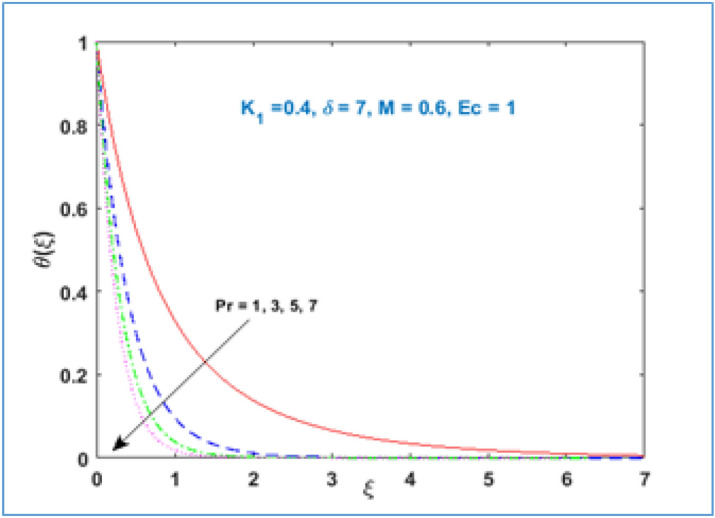
$$\theta \left(\xi \right)\;\; versus \; \; Pr.$$

Characteristic of material parameter ${K}_{1}$ on temperature profile $\theta (\xi )$ is shown in Fig. 8. It is observed the temperature profile declines with rising values of material parameter ${K}_{1}$. The effect of increasing magnetic parameter $M$ on temperature distribution is shown in Fig. 9. Here temperature profile increases as $M$ is increased. Figure 10 shows the temperature profile decreases with increment in radius of curvature parameter $\delta$. The effects of viscous dissipation or Eckert number on temperature distribution is given in Fig. 11. It is noticed that increasing values of Eckert number $Ec$ brings accelerating characteristics in temperature distribution and boundary layer thickness. Figure 12 indicates how the presence of Prandtl number effects temperature profile. The thermal boundary layer shows a diminishing trend as Prandtl number is increased. This takes place due to the fact, when the Prandtl number increases the thermal conduction of the medium decreases as a result the thermal boundary layer thickness declines.

To confirm the accuracy and validity of the employed numerical method a comparison of skin friction is made with those reported by Okechi et al.^33^ for ${K}_{1}=0, M=0$ as given in Table 1. Table 1 shows that present results agree well with the preceding data and this confirms that the numerical procedure adopted in the present work gives accurate results. Table 2 represents the behavior of skin friction against different pertinent parameter. The values of Nusselt number at the curved surface for distinct emerging non-dimensional parameters in the governing equations are given in Table 3.

**Table 1:** Values of skin friction coefficient ${-C}_{fs}$ and comparison with Okechi et al.^33^ for $\delta ,$ when ${K}_{1}$ = 0, and $M=0$.

$\delta$	SM	KBM	Okechi et al.^33^
5	1.4198	1.4198	1.4196
10	1.3468	1.3468	1.3467
20	1.3135	1.3135	1.3135
30	1.3027	1.3027	1.3028
40	1.2975	1.2975	1.2975
50	1.2944	1.2944	1.2944
100	1.2881	1.2881	1.2881
200	1.2850	1.2850	1.2850
1000	1.2825	1.2825	1.2826
$\boldsymbol{\infty }$	1.2818	1.2818	1.2818

**Table 2:** Numerical computation of skin friction coefficient $-{C}_{fs}$ and couple stress ${C}_{s}$ for different physical flow parameters.

${K}_{1}$	$M$	$\delta$	$-{C}_{fs}$		${C}_{s}$	
			SM	KBM	SM	KBM
0.4	0.5	7	1.830404	1.830427	0.1663512	0.1663536
0.6			1.989029	1.989048	0.2313259	0.2313259
1.0			2.165557	2.165570	0.361153	0.361153
0.4	0.2	7	1.737532	1.737551	0.153462	0.153464
	0.6		1.892846	1.892869	0.168982	0.168984
	1.0		2.178558	2.178580	0.192836	0.192836
0.4	0.5	10	1.781235	1.781256	0.171027	0.171030
		100	1.674426	1.674446	0.183156	0.183157
		1000	1.663331	1.663312	0.184245	0.184245

**Table 3:** Values of $\theta {^{\prime}}(0)$ for different physical flow parameters.

$\delta$	$M$	$Pr$	$Ec$	${K}_{1}$	SM	KBM
5	0.5	0.7	0.3	0.2	0.938958	0.938951
100					0.935618	0.935613
1000					0.925259	0.925259
7	0.2	0.7	0.3	0.2	0.969702	0.969696
	0.6				0.922585	0.922581
					0.849553	0.849543
7	0.5	1	0.3	0.2	1.114253	1.142527
		1.5			1.433049	1.433040
		2			1.677469	1.677457
7	0.5	0	0.6	0.2	0.812512	0.812500
			0.8		0.730346	0.730330
			1		0.648181	0.648159
				0.2	0.935760	0.935756
				0.4	0.959186	0.959184
				0.4	0.977454	0.977745

## Conclusion

In this work we have numerically studied the boundary driven flow and the heat transfer characteristics over an exponential stretchable curved wall. Solutions were obtained numerically using the shooting method and Keller box method. In the light of present work, the important findings are given below.

- The fluid velocity shows a declining behavior as magnetic parameter and radius of curvature increase.
- Increasing the material parameter results an enhancement in fluid velocity.
- As radius of curvature and magnetic parameter increase the micro-rotation profile rises from the start of the surface but opposite behavior is noticed when it is far away the surface.
- The increment in material parameter increases the micro-rotation profile.
- Temperature profile reduces with higher values of material parameter and Prandtl number whereas opposite behavior is observed for radius of curvature, magnetic parameter, and Eckert number.

The present work can be extended by taking entropy generation, moreover for other non-Newtonian fluids this work can be carry forward.
